# Virological failure, HIV-1 drug resistance, and early mortality in adults admitted to hospital in Malawi: an observational cohort study

**DOI:** 10.1016/S2352-3018(20)30172-7

**Published:** 2020-09-02

**Authors:** Ankur Gupta-Wright, Katherine Fielding, Joep J van Oosterhout, Melanie Alufandika, Daniel J Grint, Elizabeth Chimbayo, Judith Heaney, Matthew Byott, Eleni Nastouli, Henry C Mwandumba, Elizabeth L Corbett, Ravindra K Gupta

**Affiliations:** a Department of Infection and Immunity, University College London, London UK; b Department of Clinical Research, Faculty of Infectious and Tropical Diseases, London School of Hygiene & Tropical Medicine, London, UK; c Department of Infectious Disease Epidemiology, Faculty of Epidemiology and Population Health, London School of Hygiene & Tropical Medicine, London, UK; d Malawi-Liverpool-Wellcome Trust Clinical Research Programme, University of Malawi College of Medicine, Blantyre, Malawi; e Department of Medicine, University of Malawi College of Medicine, Blantyre, Malawi; f School of Public Health, University of the Witwatersrand, Johannesburg, South Africa; g Dignitas International, Zomba, Malawi; h Advanced Pathogen Diagnostics Unit, University College London Hospitals NHS Foundation Trust, London, UK; i Department of Clinical Sciences, Liverpool School of Tropical Medicine, Liverpool, UK; j Department of Medicine, University of Cambridge, Cambridge, UK; k Africa Health Research Institute, Durban, KwaZulu-Natal, South Africa

## Abstract

**Background:**

Antiretroviral therapy (ART) scale-up in sub-Saharan Africa combined with weak routine virological monitoring has driven increasing HIV drug resistance. We investigated ART failure, drug resistance, and early mortality among patients with HIV admitted to hospital in Malawi.

**Methods:**

This observational cohort study was nested within the rapid urine-based screening for tuberculosis to reduce AIDS-related mortality in hospitalised patients in Africa (STAMP) trial, which recruited unselected (ie, irrespective of clinical presentation) adult (aged ≥18 years) patients with HIV-1 at admission to medical wards. Patients were included in our observational cohort study if they were enrolled at the Malawi site (Zomba Central Hospital) and were taking ART for at least 6 months at admission. Patients who met inclusion criteria had frozen plasma samples tested for HIV-1 viral load. Those with HIV-1 RNA of at least 1000 copies per mL had drug resistance testing by ultra-deep sequencing, with drug resistance defined as intermediate or high-level resistance using the Stanford HIVDR program. Mortality risk was calculated 56 days from enrolment. Patients were censored at death, at 56 days, or at last contact if lost to follow-up. The modelling strategy addressed the causal association between HIV multidrug resistance and mortality, excluding factors on the causal pathway (most notably, CD4 cell count, clinical signs of advanced HIV, and poor functional and nutritional status).

**Findings:**

Of 1316 patients with HIV enrolled in the STAMP trial at the Malawi site between Oct 26, 2015, and Sept 19, 2017, 786 had taken ART for at least 6 months. 252 (32%) of 786 patients had virological failure (viral load ≥1000 copies per mL). Mean age was 41·5 years (SD 11·4) and 528 (67%) of 786 were women. Of 237 patients with HIV drug resistance results available, 195 (82%) had resistance to lamivudine, 128 (54%) to tenofovir, and 219 (92%) to efavirenz. Resistance to at least two drugs was common (196, 83%), and this was associated with increased mortality (adjusted hazard ratio 1·7, 95% CI 1·2–2·4; p=0·0042).

**Interpretation:**

Interventions are urgently needed and should target ART clinic, hospital, and post-hospital care, including differentiated care focusing on patients with advanced HIV, rapid viral load testing, and routine access to drug resistance testing. Prompt diagnosis and switching to alternative ART could reduce early mortality among inpatients with HIV.

**Funding:**

Joint Global Health Trials Scheme of the Medical Research Council, UK Department for International Development, and Wellcome Trust.

## Introduction

Despite the unprecedented scale-up of antiretroviral therapy (ART) in high-HIV prevalence settings in sub-Saharan Africa, HIV remains a common cause of admission to hospital, with high early mortality (31% in the African region).^1^, ^2^ While older cohorts of patients with HIV from sub-Saharan Africa were predominantly newly diagnosed or ART naive, more recent data suggests that patients admitted with advanced HIV (defined by WHO as CD4 count <200 cells per μL or stage 3 or 4 illness) are mostly ART experienced.^3^, ^4^, ^5^, ^6^

HIV drug resistance is increasingly common in sub-Saharan Africa, with recent estimates of 10–15% prevalence for resistance to non-nucleoside reverse transcriptase inhibitors (NNRTIs) in untreated patients (transmitted drug resistance), and much higher prevalence (50–80% for NNRTIs) in patients who have had ART.^7^, ^8^, ^9^ However, laboratory and clinical capacity within sub-Saharan Africa remains limited, with most countries managing patients on ART with infrequent viral load testing and minimal access to drug resistance testing. As distinguishing HIV drug resistance from alternative explanations for progressive illness on ART is usually not possible, patients who develop advanced HIV while established on ART are assumed to have treatment failure or go undetected.

**Research in context**

**Evidence before this study**

We searched MEDLINE for studies that investigated virological failure or HIV drug resistance in patients with HIV on antiretroviral therapy (ART) who are hospital inpatients in Africa, published between Jan 1, 2005, (ART was not in widespread use before this date), to Jan 1, 2020. We combined search terms for HIV (“HIV”, “HIV-1”, “Human immunodeficiency virus”, or “AIDS”) with terms for virological failure (“virological failure”, “viral failure”, “failure”, “antiretroviral failure”, “treatment failure”, or “ART failure”) or resistance (“resistance”, “drug resistance”, “HIV resistance”, “antiretroviral resistance”, or “ART resistance”), and with terms for hospital inpatient (“hospital”, “inpatient”, “in-patient”, “admission”, or “hospitalised”) and Africa. We only identified one observational cohort study that reported prevalence of virological failure in two cohorts of inpatients. This study showed a high prevalence of patients who were already taking ART but had HIV-1 viral load of at least 1000 copies per mL. However, the study did not do viral load testing on all patients taking ART, introducing bias. There were no studies reporting HIV drug resistance among hospital inpatients. Other studies identified all reported data from outpatient clinics.

**Added value of this study**

We did HIV viral load testing on unselected patients admitted to hospital who were taking ART for at least 6 months, and looked for HIV drug resistance mutations for patients who had high HIV viral loads. We then assessed whether the presence of drug resistance was associated with mortality. Approximately one-third of inpatients taking ART had virological failure, and most of these patients had drug resistance to first-line ART which was associated with increased early mortality. These are the first data on HIV drug resistance in hospital inpatients from high-burden settings in Africa that show the high prevalence of HIV drug resistance.

**Implications of all the evidence**

ART failure and drug resistance is a major problem for hospital inpatients in settings with high HIV prevalence. Our results support testing hospital inpatients for ART failure, ideally using rapid HIV-1 viral load assays so that results are available quickly and can be immediately acted upon. The high prevalence of drug resistance and association with early mortality suggests these patients should be switched to alterative ART, and supports development of low-cost, rapid assays to detect HIV drug resistance. Differentiated ART clinic care to support adherence and detect ART failure in advanced HIV, testing and screening for opportunistic infections, and improved care post discharge could improve outcomes, although further evidence for such interventions will be needed.

Although most available data on HIV drug resistance come from outpatient clinics, inpatients repwresent a key target group for intensified interventions, given their relatively high risks of treatment failure, advanced immunosuppression, and high short-term mortality.^10^ Timely diagnosis and management of ART failure and drug resistance in this patient population has the potential to improve individual patient outcomes and contribute to the UNAIDS 95-95-95 targets set for 2030.^1^ As of July 2020, few data exist describing HIV drug resistance in patients established on ART but admitted to hospital.

To describe the prevalence of virological failure and HIV drug resistance, and their effect on early mortality in patients with HIV admitted to hospital in high-HIV burden settings, we carried out an observational cohort study nested within a large tuberculosis screening trial in sub-Saharan Africa.

## Methods

### Study design

This cohort study was nested within the rapid urine-based screening for tuberculosis to reduce AIDS-related mortality in hospitalised patients in Africa (STAMP) trial, which recruited unselected (ie, irrespective of clinical presentation), adult (aged ≥18 years) patients with HIV-1 at admission to medical wards.^4^, ^11^ Enrolled patients were randomly assigned to tuberculosis screening using sputum testing alone, or sputum and urine testing. Tuberculosis screening results were provided to clinical teams, but with all patient management provided by hospital clinicians without input from study staff. All patients were managed as per Malawi national HIV guidelines (appendix p 2).

Exclusion criteria were recent tuberculosis treatment (past 12 months), recent tuberculosis preventative therapy (past 6 months), or being unable or unwilling to provide informed consent. The study team recorded clinical data at admission and during hospitalisation on standardised case report forms based on patient interview, medical records, and clinical review. Patients discharged alive were followed up at 56 days. Vital status was established by home visit, telephone, or through next of kin for those not attending follow-up.

Patients were included in this study if they were enrolled at the Malawi site (Zomba Central Hospital; appendix p 3) and were taking ART for at least 6 months at admission. We used random number generation to select a random sample of 80 patients with HIV not currently taking ART were also included to provide data on pre-treatment HIV drug resistance. Data on ART status at admission were collected by patient interview and confirmed by reviewing handheld outpatient notes (health passport) or ART prescription. At enrolment, patients had a venepuncture for CD4 cell count, haemoglobin, and plasma which was stored at −80°C.

Management of HIV infection was as per national HIV guidelines.^12^ First-line ART has been tenofovir, lamivudine, and efavirenz since 2011, and viral load monitoring was recommended routinely at 6 months, 2 years, and then twice yearly after commencing ART, and if virological failure is clinically suspected (appendix p 3). Viral load testing is not routinely done for hospital inpatients, but is mandatory before switching to second-line ART.

All patients provided informed written consent for participation and sample storage. Retrospective HIV viral load and drug resistance testing was approved by the research ethics committee of the London School of Hygiene and Tropical Medicine, and the University of Malawi College of Medicine Research and Ethics Committee. This study conforms to STROBE guidelines for observational studies (appendix p 8).

### Laboratory assessments

HIV plasma viral load was measured from frozen plasma using Abbott RealTime HIV-1 (m2000sp) viral load assay (Abbott Molecular, IL, USA) in Malawi. For enrolled patients with virological failure (defined as a plasma viral load ≥1000 copies per mL) HIV-1 genotyping to detect drug resistance mutations was done by ultra-deep sequencing in the UK.

Nucleic acids were extracted from 230 μL of plasma using the DSP Virus/Pathogen kit on the QIAsymphony platform (Qiagen, Hilden, Germany) and amplified using in-house HIV primer sets (gag-pol codons 691-3582, pol-int gag-pol codons 2696-5527, int-env [g120] gag-pol codons 5518-7374). Library preparations were generated using the Nextera XT DNA Sample Preparation Kit and sequenced on the Illumina MiSeq platform (Illumina, San Diego, CA, USA). Bioinformatic analysis was done using the de novo Iterative Virus Assembler. Following sequencing, samples were aligned using the MAFFT program (version 7).

HIV-1 subtype and drug resistance mutations were analysed using the Stanford HIVDB program (version 2.3.0).^13^ Drug resistance mutations were only considered if present in at least 20% of the viral population. The level of drug resistance was determined by adding penalty scores for each drug resistance mutation according to the Stanford HIVDB algorithm (version 8.8), with the level (1 to 5) being calculated on the basis of the total score.^14^ Drug resistance was defined as level 4 (intermediate) or level 5 (high-level) resistant. Multidrug resistance was defined as resistance to two or more first-line drugs from the first-line ART regimen.^15^

### Statistical analysis

Categorical data were compared using χ^2^ tests, continuous data using *t* tests or Wilcoxon rank sum dependent on distribution. Mortality risk was calculated 56 days from enrolment. Patients were censored at death, at 56 days, or at last contact if lost to follow-up. Cox proportional hazards models were used to assess associations with mortality, p values were calculated using likelihood ratio tests. The modelling strategy addressed the causal association between HIV multidrug resistance and mortality, excluding factors on the causal pathway (most notably, CD4 cell count, clinical signs of advanced HIV, and poor functional and nutritional status). All models were adjusted for STAMP trial arm.

In the Cox regression analysis, linear association and departures from linearity of continuous variables with log (mortality rate) were assessed using the likelihood ratio test. For the mortality regression analysis, all patients with viral load below 1000 copies per mL were assumed to have no drug resistance, and patients with virological failure but missing drug resistance data were excluded. A sensitivity analysis was done only including patients on ART for more than 12 months. Results are reported as hazard ratios (HRs) with 95% CIs and Kaplan-Meier curves. We used Stata (version 14; College Station, TX, USA) for analyses. See appendix (p 13) for statistical analysis plan.

### Role of the funding source

The funder of the study had no role in study design, data collection, data analysis, data interpretation, or writing of the report. The corresponding author had full access to all the data in the study and had final responsibility for the decision to submit for publication.

## Results

**Figure 1:**
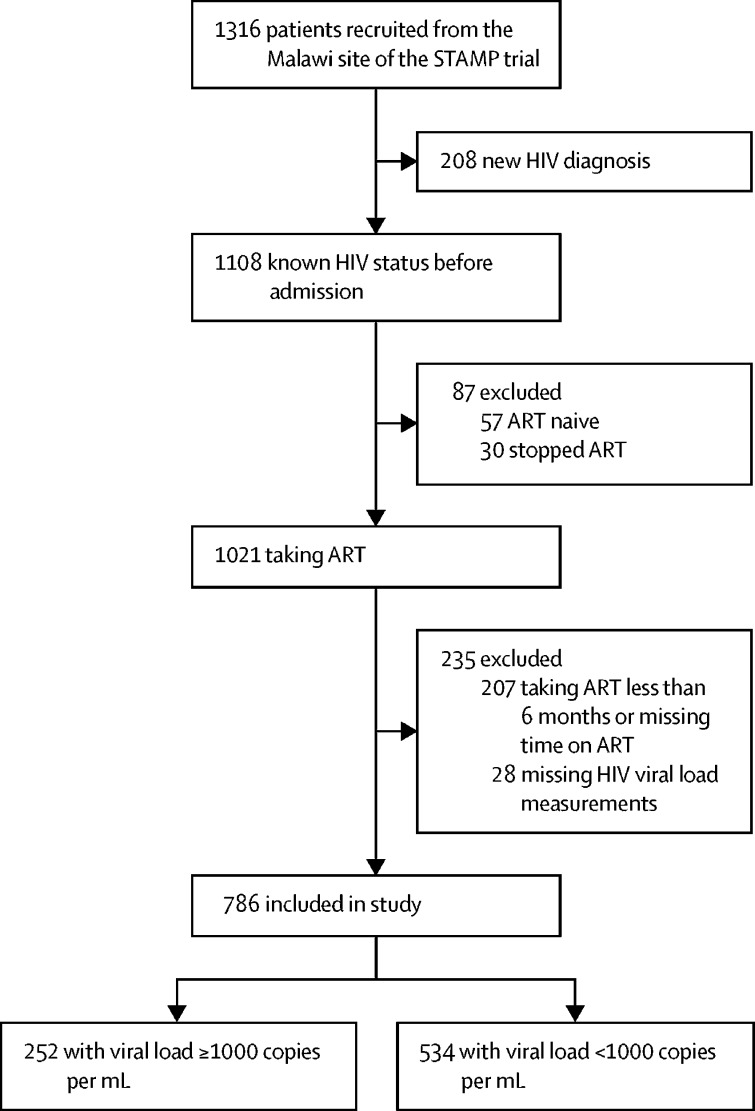
Study flow diagram ART=antiretroviral therapy.

Of 1316 patients with HIV enrolled in the STAMP trial at the Malawi site between Oct 26, 2015, and Sept 19, 2017, 1108 (84%) knew their HIV status. Of those who knew their HIV status, 1021 (92%) were taking ART, with 814 (80%) on ART for at least 6 months at hospital admission. 28 patients had missing HIV viral load measurements, leaving 786 included in the analysis (figure 1). Mean age was 41·5 years (SD 11·4), 528 (67%) of 786 were women, 770 (98%) were on first-line ART, and the median time on ART was 4·7 years (IQR 2·2–8·1). 606 (77%) had advanced HIV, defined by CD4 count below 200 cells per μL or WHO stage 3 or 4 illness (table 1).

**Table 1:** Patient characteristics at baseline, stratified by virological failure

		**Overall (n=786)**	**HIV viral load <1000 copies per mL (n=534)**	**HIV viral load ≥1000 copies per mL (n=252)**	**p value**
Age, years		41·5 (11·4)	43·1 (11·8)	38·2 (9·8)	<0·0001
Sex					0·020
	Male	258 (33%)	161 (30%)	97 (39%)	..
	Female	528 (67%)	373 (70%)	155 (62%)	..
Time on ART, years		4·7 (2·0–8·1)	4·6 (1·9–8·1)	5·0 (2·5–8·1)	0·16
ART regimen					0·64
	First line	770 (98%)	524 (98%)	246 (98%)	..
	Second line	16 (2%)	10 (2%)	6 (2%)	..
Advanced HIV*		606 (77%)	370 (69%)	236 (94%)	<0·0001
WHO tuberculosis four-symptom screening†		606 (77%)	467 (88%)	238 (94%)	0·0026
Body-mass index, kg/m^2^		19·8 (4·2)	20·5 (4·2)	18·3 (3·6)	<0·0001
WHO danger sign‡		191 (24%)	120 (23%)	71 (28%)	0·082
Karnofsky score		60 (50–70)	60 (50–70)	50 (50–60)	<0·0001
CD4 count, cells per μL§		277 (97–496)	383 (234–562)	60 (17–156)	<0·0001
HIV viral load, copies per mL		0 (0–14 782)	..	..	..
Haemoglobin, g/L		102·1 (31·0)	107·4 (31·5)	90·7 (26·8)	<0·0001

Data are mean (SD), n (%), or median (IQR). p values compare HIV viral load below 1000 copies per mL and at least 1000 copies per mL, calculated using χ^2^ tests for proportions, *t* tests for means, and Wilcoxon rank sum for medians. ART=antiretroviral therapy.

* Defined as CD4 count below 200 cells per μL or stage 3 or 4 illness.

† Defined as at least one of current cough, fever, weight loss, or night sweats.

‡ Defined as at least one of respiratory rate above 30 per minute, temperature above 39°C, heart rate above 120 beats per minute and unable to walk unaided.

§ Missing CD4 count data for two patients.

468 (60%) of 786 patients had undetectable HIV-1 viral loads (<50 copies per mL) and 252 (32%) had virological failure (viral load ≥1000 copies per mL) with a median viral load of 125 603 copies per mL. A further 66 (8%) had low-level viraemia (50–999 copies per mL). Patients with virological failure were younger, more likely than other patients to be male, and more likely to have lower body-mass index (BMI), Karnofsky scores, and CD4 cell counts (60 compared with 383 cells per μL in patients without virological failure), with 236 (94%) having advanced HIV at admission. They were also more likely to receive antimicrobial and tuberculosis treatment as inpatients, and had a longer median length of hospitalisation (17 compared with 14 days in patients without virological failure; table 2). Of the 16 patients on second-line ART, six (38%) had virological failure.

**Table 2:** Patient outcomes, stratified by virological failure

		**Overall (n=786)**	**HIV viral load <1000 copies per mL (n=534)**	**HIV viral load ≥1000 copies per mL (n=252)**	**p value**
STAMP trial arm					0·35
	Standard of care	402 (51%)	267 (50%)	135 (54%)	..
	Intervention	384 (49%)	267 (50%)	117 (46%)	..
Received any antimicrobial treatment		695 (88%)	457 (86%)	238 (94%)	0·0003
Received tuberculosis treatment		109 (14%)	66 (12%)	43 (17%)	0·075
Length of stay, days		15·0 (8·0–21·0)	14·0 (5·0–21·0)	17·0 (14·0–21·0)	0·018
Visited ART clinic after discharge		475 (67%)	312 (65%)	163 (72%)	0·074
Mortality at 56 days					
	Overall	156 (20%)	94 (18%)	62 (25%)	0·022
	During hospital admission	73 (9%)	50 (9%)	23 (9%)	0·92
	After discharge from hospital	83 (12%)	44 (9%)	39 (17%)	0·0019

Data are n (%) or median (IQR). p values compare HIV viral load below 1000 copies per mL and at least 1000 copies per mL, calculated using χ^2^ tests for proportions, *t* tests for means, and Wilcoxon rank sum for medians. ART=antiretroviral therapy.

Of 252 samples from patients with virological failure, 237 (94%) were successfully sequenced (237 had reverse transcriptase genes sequenced, 233 had protease genes sequenced, and 225 had integrase genes sequenced) and all were HIV subtype C. 221 (93%) of 237 patients had drug resistance mutations to first-line or second-line drugs.

**Figure 2:**
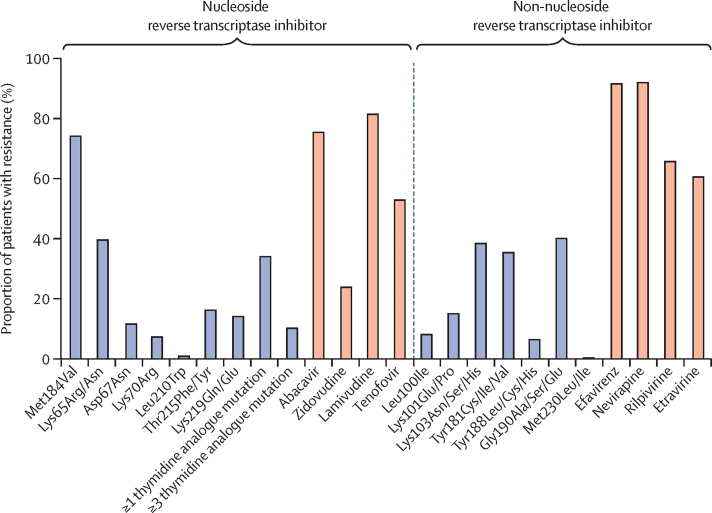
Proportion of patients with virological failure who have HIV drug resistance mutations and intermediate or high-level resistance to first-line antiretroviral therapy drugs A total of 237 samples from patients were successfully sequenced.

The most common relevant nucleoside reverse transcriptase inhibitor (NRTI) drug resistance mutations were Met184Val (75%, conferring drug resistance to lamivudine) and Lys65Arg/Asn (41%, conferring drug resistance to tenofovir; table 3; figure 2).^16^ 84 (35%) patients had at least one thymidine analogue mutation and 55 (23%) had at least three thymidine analogue mutations. Resistance to lamivudine was seen in 195 (82%) patients, and resistance to tenofovir disoproxil in 128 (54%). Resistance to other NRTIs was also common, with 181 (76%) of patients having drug resistance to abacavir and 60 (25%) to zidovudine. In 40 (17%) patients, HIV was susceptible to all NRTIs, and in 161 (68%) patients, HIV was resistant to three or all four available NRTI drugs.

**Table 3:** HIV drug resistance by ART status at admission

	**ART ≥6 months with HIV viral load ≥1000 copies per mL (n=237)**	**ART naive (n=60)**	**Previous ART (n=19)**
**Resistance to NRTIs**			
Lamivudine	195 (82%)	0	2 (11%)
Tenofovir	128 (54%)	0	2 (11%)
Abacavir	181 (76%)	0	2 (11%)
Zidovudine	60 (25%)	0	0
Stavudine	167 (71%)	0	2 (11%)
Didanosine	169 (71%)	0	2 (11%)
**Resistance to NNRTIs**			
Efavirenz	219 (92%)	7 (12%)	8 (42%)
Nevirapine	220 (93%)	7 (12%)	9 (47%)
Rilpivirine	158 (67%)	2 (3%)	5 (26%)
Etravirine	146 (62%)	2 (3%)	3 (16%)
Resistance to protease inhibitors*			
Ritonavir-boosted lopinavir	1 (<1%)	0	0
Ritonavir-boosted atazanavir	0	0	0
Ritonavir-boosted darunavir	0	0	0
**Resistance to integrase inhibitors**†			
Raltegravir	1 (<1%)	0	0
Dolutegravir	0	0	0
**Resistance to first-line ART**			
Susceptible to all drugs	18 (8%)	53 (88%)	11 (58%)
Resistance to 1 drug	23 (10%)	7 (12%)	6 (32%)
Resistance to 2 drugs	69 (29%)	0	0
Resistance to 3 drugs	127 (54%)	0	2 (11%)
Resistance to ≥2 drugs	196 (83%)	0	2 (11%)
**NRTI mutation**			
Lys65Arg/Asn	97 (41%)	0	1 (5%)
Met184Val	178 (75%)	0	2 (11%)
Leu74Ile	17 (7%)	0	0
Leu74Val	3 (1%)	0	0
**Thymidine analogue mutation**			
Met41Leu	37 (16%)	0	0
Asp67Asn	31 (13%)	0	0
Lys70Arg	21 (9%)	0	0
Leu210Trp	6 (3%)	0	0
Thr215Phe/Tyr	42 (18%)	0	0
Lys219Gln/Glu	37 (16%)	0	0
≥1 thymidine analogue mutations	84 (35%)	0	0
≥3 thymidine analogue mutations	55 (23%)	0	0
**NNRTI mutation**			
Leu100Ile	23 (10%)	0	1 (5%)
Lys103Asn/Ser/His	94 (40%)	4 (7%)	5 (26%)
Tyr181Cys/Ile/Val	87 (37%)	1 (2%)	2 (11%)
Tyr188Leu/Cys/His	19 (8%)	0	1 (5%)
Gly190Ala/Ser/Glu	98 (41%)	0	2 (11%)
Met230Leu/Ile	4 (2%)	0	0
**Protease inhibitor and integrase inhibitor mutation**‡			
Val82Ala	1 (<1%)	0	0
Gln148Gln/His	1 (<1%)	0	0
Thr66Thr/Ser	1 (<1%)	0	0

Data are n (%). ART=antiretroviral therapy. NRTI=nucleoside reverse transcriptase inhibitor. NNRTI=non-nucleoside reverse transcriptase inhibitor.

* Missing data for four patients whose protease gene was not successfully sequenced.

† Missing data for 12 patients whose integrase gene was not successfully sequenced.

‡ Missing data for 16 patients whose protease gene or integrase gene were not successfully sequenced.

NNRTI resistance was almost universal, with 219 (92%) samples resistant to efavirenz, and 220 (93%) resistant to nevirapine. The most common NNRTI mutations were Lys103Asn/Ser/His (40%), Tyr181Cys/Ile/Glu (37%), and Gly190Ala/Ser/Glu (41%). Resistance to newer NNRTI drugs was also common and consistent with anticipated cross-resistance, with 158 (67%) samples resistant to rilpivirine, and 146 (62%) resistant to etravirine.

Protease inhibitor and integrase inhibitor resistance were rare, with only one (<1%) of 233 patients having a major protease inhibitor mutation (Val82Ala), and two (1%) patients having accessory protease inhibitor mutations (Gln58Glu). Two (1%) of 225 patients also had major integrase inhibitor mutations (one had Gln148Gln/His and one had Thr66Ser), and eight (4%) had accessory integrase inhibitor mutations (six had Glu157Gln and two had Gln95Lys).

Overall, only 18 (8%) of 237 patients had no detectable resistance to any first-line ART drugs, with 127 (54%) having resistance to all first-line drugs, and 196 (83%) to at least two drugs (table 3; appendix p 5). Assuming patients with HIV viral load below 1000 copies per mL had no drug resistance, the prevalence of resistance to at least two first-line ART drugs (multidrug resistance) was 25% (196 of 771) for patients hospitalised after taking ART for six months or longer. Eight of 15 patients with viral loads between 400 and 999 copies per mL also had HIV-1 genotyping, of which seven (88%) of eight had multidrug resistance.

Mortality by day 56 was 20% (156 of 786; 95% CI 17–23), with 83 (53%) of 156 deaths occurring after discharge from hospital (table 2). Mortality was greater in those with virological failure (62 [25%] of 252) compared with those without (94 [18%] of 534; unadjusted HR 1·44, 95% CI 1·04–1·98; p=0·028). This difference mainly reflects deaths after discharge (17% [39 of 252], 95% CI 13–23 in those with virological failure *vs* 9% [44 of 534], 95% CI 7–12% in those without; p=0·0020), with inpatient mortality similar for those with virological failure (23 [9%] of 252) and those without (50 [9%] of 534).

Among 237 patients with virological failure and available HIV drug resistance genotypes, unadjusted mortality increased with increasing drug resistance. Mortality by day 56 was 6% in patients with no drug resistance, 13% in patients with resistance to one drug, and 28% in patients with multidrug resistance (p=0.041; appendix p 6).

**Figure 3:**
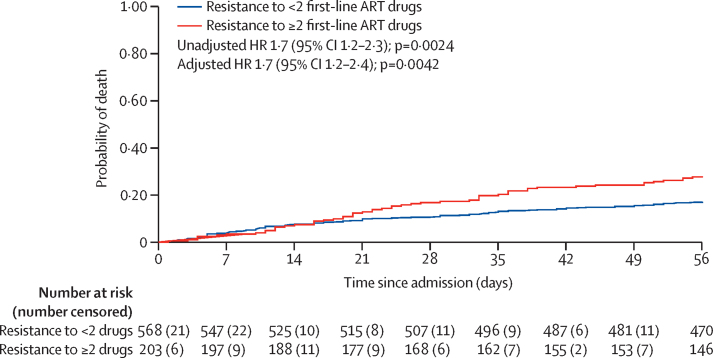
Kaplan-Meier curve showing probability of death stratified by drug resistance A total of 771 patients were included in the survival analysis. Patients with suppressed virus (<1000 copies per mL) were assumed to have no drug resistance. 15 patients without HIV drug resistance data were excluded. ART=antiretroviral therapy. HR=hazard ratio.

In analyses adjusted by STAMP trial arm only, age, sex, time on ART, advanced HIV, BMI, Karnofsky score, CD4 count, haemoglobin, WHO danger signs, tuberculosis treatment, and virological failure were all strongly associated with increased mortality (table 4). HIV multidrug resistance was associated with increased mortality (HR 1·7, 95% CI 1·2–2·3; p=0·0024) and remained so after adjustment for age, sex, time on ART, tuberculosis treatment, and trial arm (adjusted HR 1·7, 95% CI 1·2–2·4; p=0·0042; table 4; figure 3). There were no significant interactions between variables in the final model. Sensitivity analyses only including 724 patients currently taking ART for 12 months or longer yielded similar results (adjusted HR 1·9, 95% CI 1·3–2·7; p=0·0011).

**Table 4:** Univariable and multivariable Cox regression analyses of mortality at 56 days

		**Died (n=156)**	**Univariable HR for mortality (95% CI)**	**p value**	**Multivariable HR for mortality (95% CI)**	**p value**
Age, years*		43·4 (12·8)	1·02 (1–1·03)	0·022	1·02 (1·01–1·04)	0·0049
Sex						
	Male	83/258 (32%)	1 (ref)	..	1 (ref)	..
	Female	73/528 (14%)	0·39 (0·28–0·53)	<0·0001	0·49 (0·35–0·67)	<0·0001
Time on ART, years		3·9 (1·6–7·0)	0·95 (0·91–0·99)	0·023	0·94 (0·90–0·99)	0·016
ART regimen						
	First line	152/770 (20%)	1 (ref)	..	..	..
	Second line	4/16 (25%)	1·32 (0·49–3·56)	0·60	..	..
Advanced HIV†						
	No	17/180 (9%)	1 (ref)	..	..	..
	Yes	139/606 (23%)	2·61 (1·58–4·32)	<0·0001	..	..
WHO tuberculosis four-symptom screening‡						
	No	13/82 (16%)	1 (ref)	..	..	..
	Yes	143/705 (20%)	1·28 (0·73–2·26)	0·37	..	..
Body-mass index		18·3 (3·6)	0·88 (0·84–0·92)	<0·0001	..	..
WHO danger sign§						
	No	93/595 (16%)	1 (ref)	..	..	..
	Yes	63/191 (33%)	2·37 (1·72–3·26)	<0·0001	..	..
Karnofsky score		50 (40–50)	0·94 (0·93–0·96)	<0·0001	..	..
CD4 count¶		135 (42–299)	0·89 (0·86–0·93)‖	<0·0001	..	..
HIV viral load, copies per mL						
	<1000	94/534 (18%)	1 (ref)	..	..	..
	≥1000	62/252 (25%)	1·44 (1·04–1·98)	0·028	..	..
Haemoglobin, g/L		86·3 (29·3)	0·84 (0·80–0·88)**	<0·0001	..	..
Received any antimicrobial treatment						
	No	17/91 (19%)	1 (ref)	..	..	..
	Yes	139/695 (20%)	1·1 (0·67–1·83)	0·69	..	..
Tuberculosis treatment						
	No	116/677 (17%)	1 (ref)	..	1 (ref)	..
	Yes	40/109 (37%)	2·36 (1·65–3·39)	<0·0001	2·12 (1·47–3·06)	0·0002
HIV multidrug resistance^††^						
	No	100/583 (17%)	1 (ref)	..	..	..
	Yes	56/203 (28%)	1·69 (1·21–2·34)	0·0024	1·68 (1·19–2·37)	0·0042

Data are mean (SD), n/N (%), or median (IQR), unless otherwise indicated. p values were calculated using the likelihood ratio test from the Cox proportional hazards model. All models were adjusted for STAMP trial arm. There were no interactions in the adjusted models. HR=hazard ratio. ART=antiretroviral therapy.

* Unadjusted HR for a 10-year increase in age is 1·17 (95% CI 1·02–1·34), adjusted HR for a 10-year increase in age is 1·24 (95% CI 1·07–1·43).

† Defined as CD4 count below 200 cells per μL or stage 3 or 4 illness.

‡ Defined as at least one of current cough, fever, weight loss, or night sweats.

§ Defined as at least one of respiratory rate above 30 per minute, temperature above 39°C, heart rate above 120 beats per minute and unable to walk unaided.

¶ Missing CD4 count data for two patients.

‖ HR is a 50 cells per mL increase in CD4 count.

** HR is for a 10 g/L increase in haemoglobin. Defined as resistance to two or more first-line ART drugs. Missing HIV drug resistance data for 15 patients. Patients with suppressed virus (<100 copies per mL) were assumed to have no drug resistance. For all other continuous variables HR represent a one-unit increase. There was no evidence for departures from linearity for any continuous variables.

In exploratory analyses, adjusting for CD4 cell count, clinical signs of advanced HIV, and poor functional or nutritional status mitigated the association of HIV multidrug resistance and mortality, supporting their position on the causal pathway between multidrug resultant HIV and death, and their exclusion from the multivariable causal model.

79 samples from patients not taking ART at hospital admission were successfully sequenced. 60 of those patients were ART naive, and 19 had previously taken ART. Baseline characteristics differed (appendix p 7), with patients who were ART naive more likely to be male, and more likely to have less advanced HIV, higher BMI, higher Karnofsky score, and higher CD4 counts. HIV viral load was also higher in patients who were ART naive (median 603 000 copies per mL, IQR 66 600–1 300 000), and HIV drug resistance was uncommon. No NRTI drug resistance mutations were found (table 3) but seven (12%) of 60 had NNRTI resistance to efavirenz and nevirapine. Among 19 patients with previous ART exposure, two (11%) had NRTI resistance to lamivudine, tenofovir, and abacavir, and eight (42%) had resistance to efavirenz. No major mutations to protease inhibitor and integrase inhibitor were detected.

## Discussion

Our findings show that, in Malawi, most inpatients with HIV knew their HIV status and were taking ART, in contrast with previously reported hospital cohorts.^2^ However, virological failure was common (32% of patients taking ART for 6 months at admission had virological failure) and HIV drug resistance was almost universal among patients with virological failure (83% of patients were resistant to two or more ART drugs). Importantly, our data show the effect of HIV drug resistance with increased short-term mortality risk. Pre-treatment drug resistance was restricted to NNRTIs, consistent with findings from recent African community-based surveys.^7^, ^9^

To our knowledge, this is the first study to report virological failure and HIV drug resistance in unselected patients with HIV admitted to hospital in sub-Saharan Africa, and to report mortality outcomes. Hospital inpatients are an important source of information on the major causes of severe illness and death in key subpopulations such as people living with HIV. Consistent with regional HIV care cascade data, our data show undiagnosed and untreated HIV was a less common cause of severe illness than previously reported, given the high proportion of patients already taking ART at admission.

Our finding that 19% of all admissions among people living with HIV (25% of patients taking ART for any duration) had virological failure highlights the growing importance of drug resistance and system weaknesses that limit diagnosis and management of virological failure before the onset of critical illness. Other African data also show widespread ART failure among inpatients, and to a lesser extent among outpatients.^10^, ^17^ Our data showing drug resistance as an important cause of admissions and deaths are, therefore, likely to be regionally generalisable.

In our study, virological failure was synonymous with HIV drug resistance. NNRTI resistance occurred in 92% of patients, an unsurprising observation given widespread use and low barrier to resistance, but resistance was more common than in outpatient studies.^17^ We also saw high rates of resistance to newer NNRTIs (etravirine and rilpivirine), probably due to previous exposure to nevirapine.^18^ Resistance to NRTIs resistance was common, with 82% resistant to lamivudine and 54% to tenofovir (widely used in sub-Saharan Africa). The high prevalence of thymidine analogue mutations (35% had one or more, 23% had three or more) probably reflects the previous stavudine exposure. The prevalence of Lys74Ile as a compensatory mutation for Met184Val/Ile also suggests that first-line ART had been failing in these individuals for a substantial period of time.^19^ Although protease inhibitors and integrase inhibitors are included in alternative ART regimens,^20^ the lack of major resistance to these drug classes reflects little drug exposure and high barriers to resistance. The low prevalence of pre-treatment HIV drug resistance implies acquisition of drug resistance during treatment, most likely driven by suboptimal adherence. We also found multidrug resistance in patients with low-level viraemia, the clinical significance of which remains unclear.

Mortality in this cohort was high: 20% patients died by day 56. We found markers of advanced HIV disease were associated with mortality. HIV drug resistance was also associated with mortality, showing a dose-response relationship, and multidrug resistance was independently associated with mortality. Viral load testing is rarely done for inpatients both generally and in Malawi specifically, and centralised testing programmes tend to have long turnaround times and lack electronic laboratory management systems, causing patients to either have died or been discharged before results are available.^21^ Therefore, virological failure in this study would have gone undetected and untreated.

Our findings suggest interventions aimed at preventing and diagnosing virological failure and drug resistance could reduce morbidity and mortality in patients taking ART. Before hospital admission, patients were actively attending ART clinics that did not identify and address their ART failure and advanced HIV. Although the public health approach to ART has led to declines in HIV incidence and mortality, patients with advanced disease (despite engagement with clinics) would benefit from differentiated care, with a focused approach to patients with high viral loads and more frequent viral load testing, review, and monitoring.^22^, ^23^ This might be challenging, given the increases in task shifting and increasingly complex ART regimens and interactions, although clinical decision tools could help identify patients at high risk of poor outcomes.^24^

Currently, HIV drug resistance testing has not been prioritised by HIV programmes in high-burden settings in sub-Saharan Africa. Point-of-care technologies to detect important drug resistance mutations are in the pipeline, and their use to guide ART choice has led to improved outcomes among patients with pre-treatment drug resistance before initiation of first-line ART.^25^, ^26^ Our findings should encourage investment in resistance testing for patients with repeat high viral loads.

In hospital, patients taking ART who have signs of advanced HIV would benefit from rapid, near-patient testing for virological failure, for example using the Xpert HIV-1 viral load assay (Cepheid, Sunnyvale, CA, USA). Its use was recently shown to increase viral suppression and retention in community-based care in South Africa.^27^ Patients identified as having ART failure will need switching to alternative regimens with adherence support, to prevent mortality. Screening and empirical treatment for opportunistic infections is also recommended by WHO advanced HIV guidelines.^5^ Post-hospital care, especially for patients with ART failure, should be improved. Systems to ensure post-discharge follow-up and monitoring of patients with ART failure would help reduce the high mortality seen in the weeks following discharge.

Malawi and WHO guidelines for ART introduced dolutegravir-based regimens (with tenofovir and lamivudine NRTI backbone) as the preferred first-line ART from 2019.^20^, ^28^ This has important implications for our findings, as dolutegravir will likely overcome the high levels of NNRTI resistance, has high barriers to resistance, and can be co-administered with tuberculosis treatment.^29^, ^30^ However, the high prevalence of NRTI resistance (68% of patients in our study whose samples were successfully sequenced were resistant to three or more NRTIs) suggests a substantial number of patients could be on functional dolutegravir monotherapy. Data from the EARNEST trial^31^ has shown that even in the presence of predicted NRTI resistance, viral suppression with protease inhibitor and NRTI-based regimens was good and 89% of patients had viral suppression. However, these patients were not acutely unwell, and recent data suggest that dolutegravir might be less potent than previously expected in patients with high viral loads,^32^ and have a lower barrier to resistance compared with ritonavir-boosted protease inhibitors when used as monotherapy.^33^ The real-world outcomes from dolutegravir-based ART and the effect of HIV multidrug resistance therefore remains to be established.

The strengths of our study are that it is a large cohort nested in a clinical trial, and unselected patients with HIV were enrolled, reducing bias. Furthermore, the data reflect routine clinical care in hospitalised patients. There are also some limitations. It is a single-centre study, although results are likely to be generalisable to other settings in sub-Saharan Africa. There is also missing data on HIV viral loads and HIV drug mutations. The exclusion of patients not consenting might have underestimated virological failure and multidrug resistance, as these people are likely to be more unwell. We assumed that patients with viral loads below 1000 copies per mL had no drug resistance, but the prevalence and implications of resistance in this group is not clear. We did not have detailed adherence information for patients. However, patients all self-reported taking ART and had evidence of attending ART clinics and ART being dispensed. Furthermore, high prevalence of drug resistance also supports that patients were taking drugs, as reversion to wild type is common without drug pressure.

In conclusion, we have found virological failure and HIV drug resistance to be extremely common in inpatients with HIV, and drug resistance was associated with increased mortality. Patients already established on ART with advanced HIV disease need screening for failure during hospital admission, ideally using rapid assays. Those identified as having ART failure would likely benefit from switching to alternative ART (based on integrase or protease inhibitors, given NNRTI and NRTI resistance). Interventions targeting ART clinic and post-hospital care are also needed. Our findings support the development of low-cost and rapid assays to detect HIV drug resistance.
