# Supplementary material for: Virological failure, HIV-1 drug resistance, and early mortality in adults admitted to hospital in Malawi: an observational cohort study
Source: Lancet HIV. 2020 Sep 2;7(9):e620–8. doi: 10.1016/S2352-3018(20)30172-7 (PMC7487765; doi:10.1016/S2352-3018(20)30172-7)
Supplement: Supplementary appendix [file mmc1.pdf]

THE LANCET
HIV

Supplementary appendix

This appendix formed part of the original submission and has been peer reviewed. We post it as supplied by the authors.

Supplement to: Gupta-Wright A, Fielding K, van Oosterhout JJ, et al. Virological failure, HIV-1 drug resistance, and early mortality in adults admitted to hospital in Malawi: an observational cohort study. *Lancet HIV* 2020; **7:** e620–28.

Lancet HIV Supplementary Appendix

<u>Virological failure, HIV-1 drug resistance and early mortality in adults admitted to hospital in Malawi: an observational cohort study</u>

Table of Contents

Supplementary methods *3*

Figure S1: Number and overlap of patients with virological failure who have intermediate or high level resistance to first-line ART drugs *5*

Figure S2: Proportion of patients on ART for over 6 months with virological failure who died or survived by 2-months, stratified by drug resistance (n=237). *6*

Table S1: Patient characteristics at baseline, stratified by antiretroviral therapy status *7*

STROBE Statement *8*

Statistical Analysis Plan *13*

References *17*

Supplementary methods

Study setting:
Zomba Central Hospital, Southern Malawi

Zomba Central hospital: Zomba Central Hospital is one of four central hospitals in Malawi and serves as a district hospital within Zomba district and a tertiary referral centre to 4 other district hospitals. The hospital has 500 beds and 4 main clinical departments: internal medicine, surgery, paediatrics and obstetrics & gynaecology. There is no emergency department, admissions are made through outpatient clinics. The hospital is mostly staffed by clinical officers (having undertaken a 3-year Diploma in Clinical medicine). All routine care is provided free of charge to the user.

Location and epidemiology: Zomba is a largely rural district in Southern Malawi with an estimated population of 799,000 in 2015, of whom 138,000 live in Zomba City. HIV prevalence is estimated at 16.8% among 15-49 year olds in 2015. Zomba is one of the highest TB burden districts in Malawi, and notification rates were estimated at 150 per 100,000 in 2015. Over half of the TB notifications occur in Zomba Central Hospital.

Management of HIV-1 and antiretroviral therapy
Malawi implemented a universal 'test and treat' strategy in 2016, with HIV care delivered through ART clinics at local health centres. Standard first-line ART for adults consisted of tenofovir disoproxil fumarate (TDF), lamivudine (3TC) and efavirenz (EFV) as a fixed-dose combination tablet since 2011.[1] Alternative first line regimens include abacavir (ABC) or zidovudine (AZT) with 3TC as nucleoside reverse transcriptase inhibitor (NRTI) backbone, and either EFV or nevirapine (NVP) as the third, non-nucleoside reverse transcriptase inhibitor (NNRTI) drug. Second-line therapy was either TDF/3TC, AZT/3TC or ABC/3TC NRTI backbone with ritonavir-boosted protease inhibitor (PI); atazanavir (ATV/r) or lopinavir (LPV/r).

Routine viral load testing was recommended routinely at 6 months, 2 years and then 2 yearly after commencing ART. If patients are clinically suspected of ART failure (ie on ART for >12 months and has new HIV-related disease, unexplained weight loss), they should undergo targeted viral load testing if good adherence in the last three months. Viral load testing is mandatory before switching to second line ART. Indications, interpretation, and actions for viral load testing in Malawi is shown in figure 1.

Programmatic HIV viral load testing uses dried blood spots, with testing done at centralised laboratories (including a laboratory at Zomba Central Hospital). At the time of the study, turnaround times for programmatic HIV viral load test results was often 4 to 8 weeks. Nationally viral load testing coverage has gradually improved during the study period (39% in October 2015, 57% in September 2017).

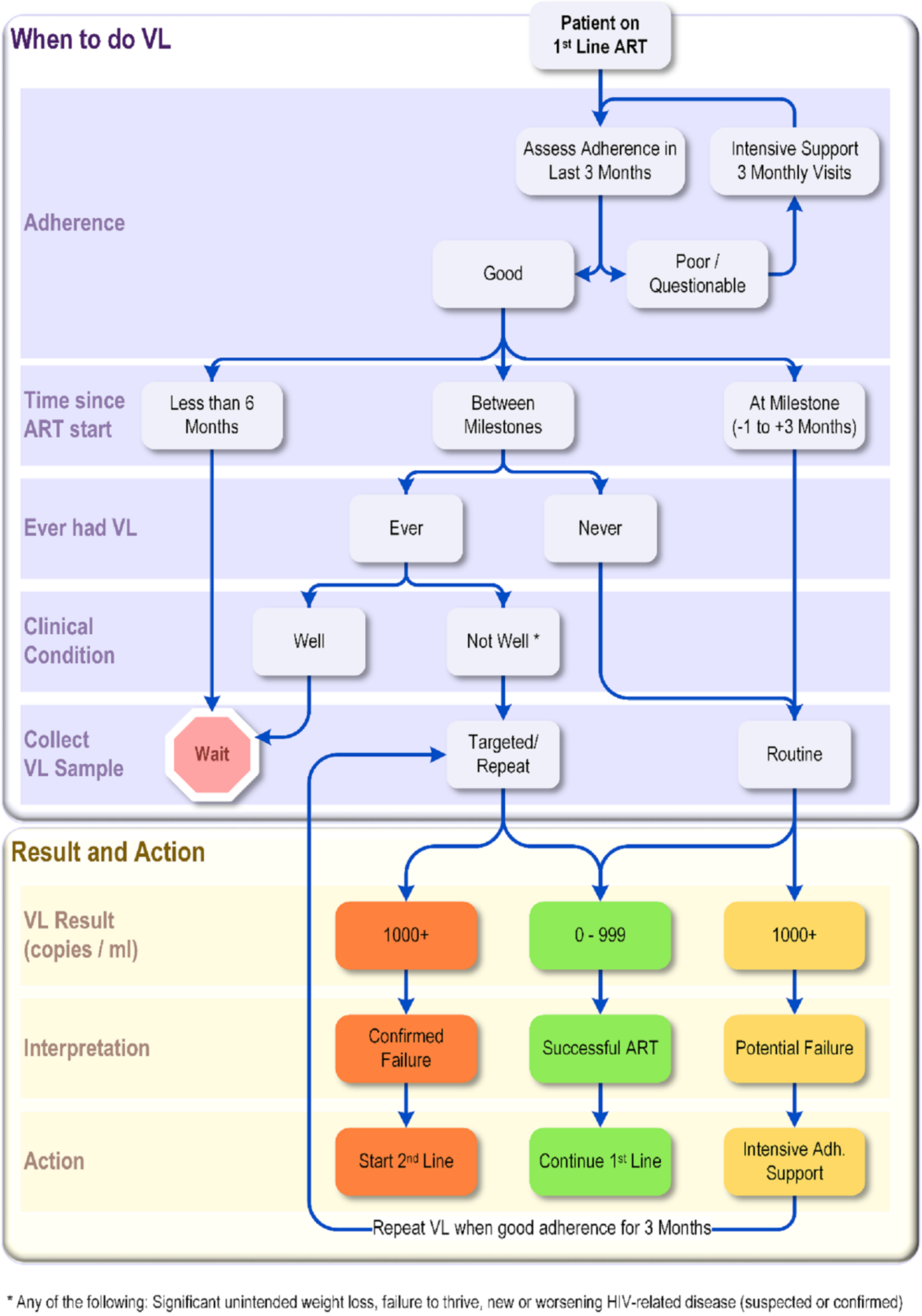

Indication, interpretation and action for routine scheduled and targeted viral load testing. Taken from the 3rd Edition of the Malawi Guidelines for Clinical Management of HIV in Children and Adults (May 2016)[1]

Figure S1: Number and overlap of patients with virological failure who have intermediate or high level resistance to first-line ART drugs

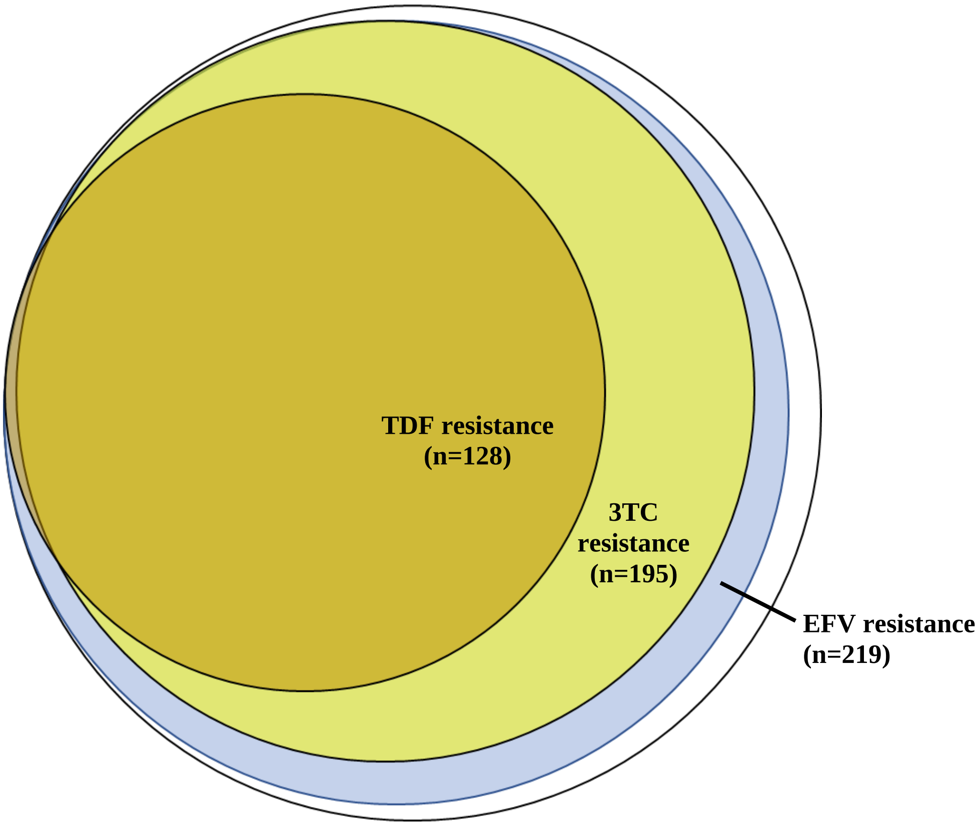

N=237, ART antiretroviral therapy; VL viral load; EFV efavirenz; 3TC lamivudine; TDF tenofovir.

<u>Figure S2: Proportion of patients on ART for over 6 months with virological failure who died or survived by 2-months, stratified by drug resistance (n=237).</u>

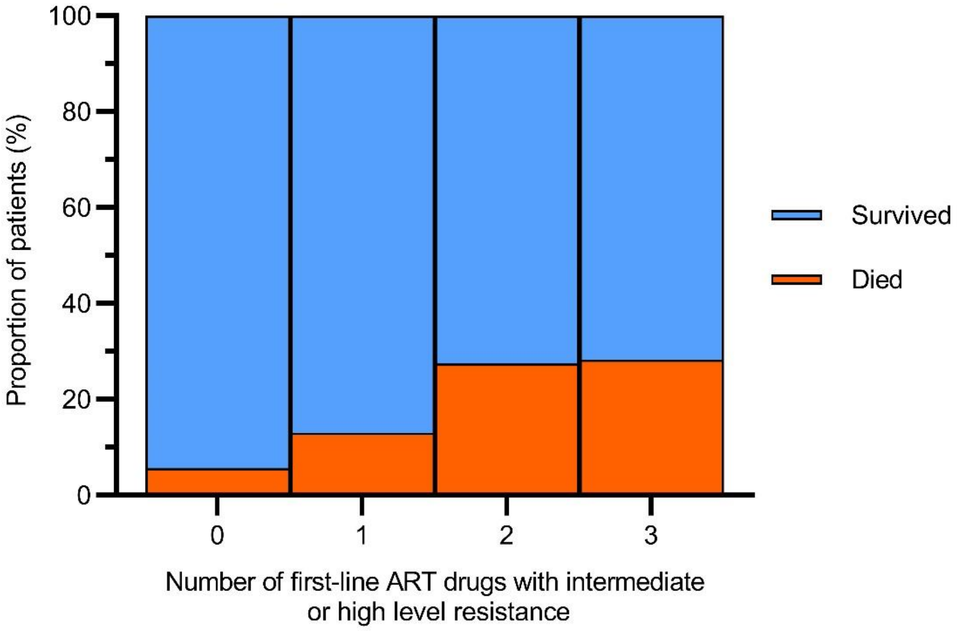

The proportion of patients with viral loads >1000 copies/mL who survived or died by 56-days stratified by number of first-line ART drugs to which there was resistance, n=18 for no resistance, n=23 for resistant to 1 drug, n=69 for resistance to 2 drugs and n=127 for resistance to 3 drugs (total n=237). P-value using chi-squared p=0.041. ART antiretroviral therapy

Table S1: Patient characteristics at baseline, stratified by antiretroviral therapy status

		Taking ART for ≥6 months N=786	ART Naive N=60	ART prior experience N=19
**Age (years)**	mean (SD)	41.5 (11.4)	37.5 (10.7)	37.6 (11.8)
**Sex**	Male	258 (32.8%)	29 (48.3%)	9 (47.4%)
	Female	528 (67.2%)	31 (51.7%)	10 (52.6%)
**Time on ART**	median (IQR)	4.9 (2.2, 8.1)	N/A	3.0 (1.3, 5.0)
**ART regimen**	1st Line	770 (98.0%)	N/A	N/A
	2nd line	16 (2.0%)		
**Advanced HIV**	No	180 (22.9%)	11 (18.3%)	1 (5.3%)
	Yes	606 (77.1%)	49 (81.7%)	18 (94.7%)
**Body Mass Index (BMI)**	mean (SD)	19.8 (4.2)	20.4 (3.3)	19.2 (3.2)
**WHO Danger sign**	Yes	191 (24.3%)	20 (33.3%)	7 (36.8%)
**Karnofsky score**	median (IQR)	60 (50, 70)	60 (50, 70)	60 (50, 70)
**CD4 count**	median (IQR)	277 (97, 496)	112.0 (39, 267)	154 (38, 310)
**HIV viral load (copies/ml)**	median (IQR)	0 (0, 14782)	603000 (66600, 1300000)	95900 (55200, 181300)
**Haemoglobin (g/l)**	mean (SD)	102.1 (31.0)	95.3 (30.3)	93.5 (23.1)

ART Antiretroviral therapy; WHO World Health Organization; SD standard deviation; IQR interquartile range. Advanced HIV defined as CD4 count <200 cells/µL or stage 3 or 4 illness. WHO 4 symptom TB screen is defined as ≥1 of current cough, fever, weight loss or night sweats WHO Danger sign is as ≥1 of respiratory rate >30 per minute, temperature >39°C, heart rate >120 beats per minute and unable to walk unaided.

STROBE Statement

Virological failure, HIV-1 drug resistance and early mortality in adults admitted to hospital in Malawi: a nested observational cohort study

	Item No.	Recommendation	Page No.	Relevant text from manuscript
**Title and abstract**	1	(*a*) Indicate the study's design with a commonly used term in the title or the abstract	title	nested observational cohort study
		(*b*) Provide in the abstract an informative and balanced summary of what was done and what was found	abstract	
		**Introduction**		
Background/rationale	2	Explain the scientific background and rationale for the investigation being reported	Introduction, paragraph 1-3	
Objectives	3	State specific objectives, including any prespecified hypotheses	Introduction, paragraph 4	To describe the prevalence of virological failure and HIV drug resistance, and their impact on early mortality in HIV-positive patients admitted to hospital in high-HIV burden settings
		**Methods**		
Study design	4	Present key elements of study design early in the paper	Methods, paragraph 1-3	
Setting	5	Describe the setting, locations, and relevant dates, including periods of recruitment, exposure, follow-up, and data collection	Methods, paragraph 1-3	Dates in results
Participants	6	(*a*) *Cohort study*—Give the eligibility criteria, and the sources and methods of selection of participants. Describe methods of follow-up<br>*Case-control study*—Give the eligibility criteria, and the sources and methods of case ascertainment and control selection. Give the rationale for the choice of cases and controls<br>*Cross-sectional study*—Give the eligibility criteria, and the sources and methods of selection of participants	Methods, paragraph 1-3	Inclusion/exclusion for parent Trial and this cohort study
		(*b*) *Cohort study*—For matched studies, give matching criteria	NA	

		and number of exposed and unexposed<br>*Case-control study*—For matched studies, give matching criteria and the number of controls per case		
Variables	7	Clearly define all outcomes, exposures, predictors, potential confounders, and effect modifiers. Give diagnostic criteria, if applicable	4-5	Mostly standard measurements, definitions and details given where necessary
Data sources/ measurement	8*	For each variable of interest, give sources of data and details of methods of assessment (measurement). Describe comparability of assessment methods if there is more than one group	Methods, 'Study setting, design and procedures'; Laboratory methods and HIV genotyping paragraph 1 and 3, 'Statistical analysis'	
Bias	9	Describe any efforts to address potential sources of bias	Methods- 'Statistical analysis'	See statistical methodology
Study size	10	Explain how the study size was arrived at	S2 appendix (SAP)	No formal sample size calculations done due to nested observational nature of study

Continued on next page

Quantitative variables	11	Explain how quantitative variables were handled in the analyses. If applicable, describe which groupings were chosen and why	Methods- ‘Statistical analysis’, and S2 appendix (SAP)	
Statistical methods	12	(*a*) Describe all statistical methods, including those used to control for confounding	Methods- ‘Statistical analysis’ and S2 appendix (SAP)	
		(*b*) Describe any methods used to examine subgroups and interactions	Methods- ‘Statistical analysis’ and S2 appendix (SAP)	Interactions were assessed using likelihood ratio testing
		(*c*) Explain how missing data were addressed	Methods- ‘Statistical analysis’ and S2 appendix (SAP)	
		(*d*) *Cohort study*—If applicable, explain how loss to follow-up was addressed<br>*Case-control study*—If applicable, explain how matching of cases and controls was addressed<br>*Cross-sectional study*—If applicable, describe analytical methods taking account of sampling strategy	S2 appendix (SAP)	Patients censored at date last seen for patients LTFU
		(*e*) Describe any sensitivity analyses	Methods- ‘Statistical analysis’ and S2 appendix (SAP)	
**Results**				
Participants	13*	(a) Report numbers of individuals at each stage of study—eg numbers potentially eligible, examined for eligibility, confirmed eligible, included in the study, completing follow-up, and analysed	Results paragraph 1, figure 1	See study flow diagram (fig 1)
		(b) Give reasons for non-participation at each stage	Results paragraph 1, figure 1	See study flow diagram (fig 1)
		(c) Consider use of a flow diagram	figure 1	See study flow diagram (fig 1)
Descriptive data	14*	(a) Give characteristics of study participants (eg demographic, clinical, social) and information on exposures and potential confounders	Results paragraph 1, table 1	Table 1
		(b) Indicate number of participants with missing data for each variable of interest	Table 1 and 3	
		(c) *Cohort study*—Summarise follow-up time (eg, average and total amount)	NA	
Outcome data	15*	*Cohort study*—Report numbers of outcome events or summary measures over time	Results paragraph 2,3, 4, 7, 9	
		Case-control study—Report numbers in each exposure category, or summary measures of exposure		

		Cross-sectional study—Report numbers of outcome events or summary measures		
Main results	16	(*a*) Give unadjusted estimates and, if applicable, confounder-adjusted estimates and their precision (eg, 95% confidence interval). Make clear which confounders were adjusted for and why they were included	Results ‘mortality’, table 3	
		(*b*) Report category boundaries when continuous variables were categorized	NA	
		(*c*) If relevant, consider translating estimates of relative risk into absolute risk for a meaningful time period	NA	All estimates as absolute risks

Continued on next page

Other analyses	17	Report other analyses done—eg analyses of subgroups and interactions, and sensitivity analyses	Results ‘mortality’
**Discussion**			
Key results	18	Summarise key results with reference to study objectives	Discussion paragraph 1
Limitations	19	Discuss limitations of the study, taking into account sources of potential bias or imprecision. Discuss both direction and magnitude of any potential bias	Discussion, penultimate paragraph
Interpretation	20	Give a cautious overall interpretation of results considering objectives, limitations, multiplicity of analyses, results from similar studies, and other relevant evidence	Discussion (throughout), discussion last paragraph
Generalisability	21	Discuss the generalisability (external validity) of the study results	Discussion, penultimate paragraph
**Other information**			
Funding	22	Give the source of funding and the role of the funders for the present study and, if applicable, for the original study on which the present article is based	Funding statement

*Give information separately for cases and controls in case-control studies and, if applicable, for exposed and unexposed groups in cohort and cross-sectional studies.

Note: An Explanation and Elaboration article discusses each checklist item and gives methodological background and published examples of transparent reporting. The STROBE checklist is best used in conjunction with this article (freely available on the Web sites of PLoS Medicine at http://www.plosmedicine.org/, Annals of Internal Medicine at http://www.annals.org/, and Epidemiology at http://www.epidem.com/). Information on the STROBE Initiative is available at www.strobe-statement.org.

Statistical Analysis Plan

Virological failure, HIV drug resistance and early mortality in adults admitted to hospital in Malawi: nested retrospective observational cohort study from the STAMP trial

Statistical Analysis Plan (version 1.0, 4th July 2019)

Introduction and background

Despite scale-up of ART in the sub-Saharan Africa, antiretroviral therapy (ART) failure appears common in patients admitted to medical wards in high HIV-prevalence settings, based on recent data from the STAMP trial and two MSF hospital cohorts [2,3]. However, the prevalence of confirmed virological failure, HIV drug resistance and associations with mortality are not well described in hospitalised patients in the era of widespread access to ART.

Aim

1. To describe the prevalence of virological failure and HIV drug resistance in patients admitted to hospital who have been established on ART.
2. Describe the association between HIV drug resistance and mortality, establish if HIV drug resistance is associated with mortality.

Study population

The population in this study will be those patients enrolled in the STAMP trial and recruited at the Zomba Central Hospital site, who are established on ART prior to admission.

Inclusion criteria

- Enrolled in Zomba (Malawi) site of the STAMP trial
- At admission, report currently taking ART
- ART start date at least 6 months prior to admission

Exclusion criteria

- Report having stopped ART
- ART start date <6 months prior to admission
- Missing ART start date

Patients fulfilling the inclusion/exclusion criteria will have stored plasma (taken at admission/enrolment) tested for HIV-1 RNA using qualitative PCR assay. Virological failure will be defined as viral load of ≥1000 copies/ml. Patients with virological failure, as described above, will then have HIV-1 genotyping for HIV drug resistance mutations. A control group of 80 patients not currently taking ART will also have stored plasma samples undergo HIV-1 genotyping for HIV drug resistance mutations to describe prevalence of pre-treatment (or transmitted) drug resistance. Some of these patients will have previously taken ART and then stopped. These patients will be chosen at random from a list of eligible patient identification numbers.

Sample size was based on patients already recruited. Prior to this study, it was known that 1021/1316 (77.6%) of patients recruited for STAMP in Malawi were currently taking ART, for a median of 3.4 years. Assuming 75% of these patients were on ART >6months, it was estimated that approximately 750 patients would require HIV viral load testing for diagnosis of virological failure of ART.

Definitions

Virological failure is defined as HIV viral load ≥1000 copies/ml. Virological suppression will be defined as undetectable HIV-1 virus (ie below the low limit of detection of the assay, <50 copies/ml). HIV virus will be considered resistant to a drug if HIV drug resistance mutations lead to level 4 (intermediate) or level 5 (high-level) resistance as per the Stanford HIVdb algorithm. Multidrug resistance will be defined as resistance to two or more drugs from the first line ART regimen (lamivudine, tenofovir, or one of efavirenz or nevirapine).

Methods: virological failure and drug resistance

- Categorical data will be compared using chi-square tests or Fischer's Exact test, continuous data using t-tests or Wilcoxon rank sum dependent on distribution
- Baseline characteristics, including relevant demographic and clinical variables (see appendix) will be described for the overall cohort, and those with and without virological failure, comparing groups using appropriate statistical tests
- Patients established on ART but missing data on HIV viral load will be excluded.
- The prevalence of important drug resistance mutations will be reported, stratified by ART status (currently taking ART, and control group (ART naïve/prior exposure to ART)
- The number of patients with missing data on HIV drug resistance mutations will be reported, and only complete cases will be included
- The prevalence of resistance to commonly used or relevant HIV drugs will be reported stratified by ART status, grouped by drug class: nucleoside reverse transcriptase inhibitor (NRTI), non-nucleoside reverse transcriptase inhibitor (NNRTI), protease inhibitor (PI) or integrase inhibitor (INI)
- The number of HIV drugs to which there was resistance, and proportion of patients with multidrug resistance will be reported

Methods: mortality

- Mortality was measured as the primary outcome of the STAMP trial at 56-days
- In-patient (death before discharge) and outpatient (death after discharge) mortality will also be reported
- For mortality analyses, all patients with viral load <1000 copies/ml will be assumed to have no drug resistance, and patients with virological failure but missing drug resistance data will be excluded from relevant analysis (if over 10% missing HIV drug resistance data, multiple imputation will be considered)

- Proportion who died by 56-days will be reported for the overall cohort, and stratified by virological failure
- Mortality will be reported for patients with and without multidrug resistance, and by number of drugs to which there was resistance (restricted to patients with data on HIV drug resistance)
- Mortality risk will be calculated up to 56 days from enrolment, patients will be censored at death, 56-days or at last contact if lost to follow-up.
- Cox proportional hazards will be used to assess associations between variables and mortality, with the variable of interest being HIV multidrug resistance.
- Unadjusted analyses will be done between relevant baseline variables and mortality, reporting hazard ratios and 95% confidence intervals. P-values will be calculated using Wald or likelihood ratio tests.
- Association between STAMP trial randomisation arm and mortality will be assessed, and models adjust for randomisation arm as appropriate
- A multivariable causal model will be built to assess the association of HIV multidrug resistance with mortality. Variables on the causal pathway between HIV drug resistance and death will be excluded (based on prior knowledge and/or relationships between variable and HIV multidrug resistance, and variable and mortality).
- Continuous variables will be assessed for departures from the linearity through categorising variables and using likelihood ratio testing to compare categorical and linear models. For variables not showing linearity, fractional polynomials will be used to help categorisation.
- Interactions will be assessed using likelihood ratio testing to compare models
- Kaplan-Meier curves and risk tables will be produced for patients with/without HIV multidrug resistance.

Ethical considerations

Ethical approval for retrospective HIV viral load and drug resistance testing has given by the research ethics committee of the London School of Hygiene and Tropical Medicine, and the University of Malawi College of Medicine Research and Ethics Committee (COMREC).

**Category**	**Variable**	**Comment**
**Demographics**	Age	
	Gender	
**Admission**	New diagnosis	Yes/No
	ART regimen	
	Time on ART	
	Presence of TB symptoms /WHO TB symptom screen	Cough, fever, night sweats, weight loss
	TB treatment	
	Advanced HIV	
	Karnofsky score/assessment of function	
	WHO danger signs	
	Weight/BMI	
**Lab results (at admission)**	CD4 cell count (absolute)	
	Haemoglobin	
	HIV viral load	
**Treatments/outcomes**	TB treatment	
	Anti-microbial	
	Mortality	
	Length of stay (days)	

References

1 Ministry of Health Malawi. Malawi Guidelines for Clinical Management of HIV in Children and Adults, 3rd Edition. 2016 http://www.moh.gov.bw/Publications/Handbook_HIV_treatment_guidelines.pdf.

2 Gupta-Wright A, Corbett EL, van Oosterhout JJ, *et al.* Rapid urine-based screening for tuberculosis in HIV-positive patients admitted to hospital in Africa (STAMP): a pragmatic, multicentre, parallel-group, double-blind, randomised controlled trial. *Lancet* 2018; **392**: 292–301.

3 Ousley J, Niyibizi AA, Wanjala S, *et al.* High Proportions of Patients with Advanced HIV Are Antiretroviral Therapy Experienced: Hospitalization Outcomes from 2 Sub-Saharan African Sites. *Clin Infect Dis* 2018; **66**: S126–32.
